# Engaging public health decision makers in partnership research

**DOI:** 10.1186/1748-5908-10-S1-A80

**Published:** 2015-08-20

**Authors:** Maureen Dobbins, Robyn Traynor

**Affiliations:** 1 Health Evidence, School of Nursing, Faculty of Health Sciences, McMaster University, Hamilton, ON L8P 0A1, Ontario, Canada

## Objective

Involving decision makers in collaborative research partnerships can help increase the relevance and timeliness of the research question, and ensure the results are more readily applied in practice. These partnerships offer great benefits but also unique challenges. We will discuss some of these challenges, as identified from our recent study (Canadian Institutes of Health Research FRN 101867, 126353) and the growing literature on engaging decision-makers in knowledge translation (KT) research. We will also recommend strategies for ensuring a successful partnership.

## Methods

We collaborated with three Canadian public health departments to enhance capacity for and facilitate organizational contexts conducive to evidence-informed decision making (EIDM). The research team and decision-maker partners jointly developed the research questions and KT strategies, tailored to each partner's organizational needs and goals. Intervention effectiveness was assessed via quantitative (online survey, in-person assessment) and qualitative (interviews, reflective journal entries, case study notes) data; this discussion has been informed, in part, by the qualitative analysis.

## Results

Identified challenges include: unpredictable practice settings and a change in priorities over time; time and staff workload; decision maker research knowledge and prior experience; and balancing applied research with rigorous scientific practice. To mitigate these challenges, we recommend: sustaining open and ongoing communication to maintain momentum and reinforce commitment; identifying a key contact to help facilitate and promote the study; obtaining formal approval on timelines, goals, communication, and role expectations; and establishing a mutual understanding of the research and decision-making processes.

## Conclusions

A strong relationship between researchers and decision makers, built on mutual respect, trust, and understanding, is critical for successful partnerships. This paradigm is continuing to gain recognition as an effective approach to KT research. Our discussion on possible challenges (and their associated solutions) will help ensure both researchers and decision makers are able to enter more productive partnerships.

